# Luteolin Inhibits Human Keratinocyte Activation and Decreases NF-κB Induction That Is Increased in Psoriatic Skin

**DOI:** 10.1371/journal.pone.0090739

**Published:** 2014-02-28

**Authors:** Zuyi Weng, Arti B. Patel, Magdalini Vasiadi, Anastasia Therianou, Theoharis C. Theoharides

**Affiliations:** 1 Molecular Immunopharmacology and Drug Discovery Laboratory, Department of Integrative Physiology and Pathobiology, Tufts University School of Medicine, Boston, Massachusetts, United States of America; 2 Graduate Program in Pharmacology and Experimental Therapeutics, Sackler School of Graduate Biomedical Sciences, Tufts University, Boston, Massachusetts, United States of America; 3 Graduate Program in Biochemistry, Sackler School of Graduate Biomedical Sciences, Tufts University, Boston, Massachusetts, United States of America; 4 First Department of Dermatology, A. Sygros Hospital, Athens University Medical School, Athens, Greece; 5 Department of Internal Medicine, Tufts University School of Medicine and Tufts Medical Center, Boston, Massachusetts, United States of America; University of Tennessee, United States of America

## Abstract

Psoriasis (Ps) is an autoimmune disease characterized by keratinocyte hyperproliferation and chronic inflammation, with increased expression of tumor necrosis factor (TNF) and vascular endothelial growth factor (VEGF). Anti-TNF biologic agents are effective in treating Ps, but are associated with increased risk of infections and blood malignancies. Moreover, keratinocyte hyperproliferation and activation have yet to be addressed. Flavonoids, such as luteolin, are natural compounds with potent anti-inflammatory properties, but their actions on keratinocytes remain unknown. We show that TNF (50 ng/mL) triggers significant production of inflammatory mediators interleukin-6, interleukin-8 and VEGF from both human HaCaT and primary keratinocytes. Pretreatment with the flavonoid luteolin (10–100 µM) significantly inhibits mRNA expression and release of all three mediators in a concentration-dependent manner. More importantly, luteolin decreases TNF-induced phosphorylation, nuclear translocation and DNA binding of the nuclear factor-kappa B (NF-κB) typically involved in inflammatory mediator transcription. We also report that luteolin reduces TNF-induced mRNA expression of two genes (NFKB1 and RELA) encoding two NF-κB subunits (NF-κB p50 and NF-κB p65, respectively). Interestingly, we show that gene expression of RELA is increased in human psoriatic skin. Keratinocyte proliferation, which is a characteristic feature of psoriatic skin, is effectively reduced by luteolin in HaCaT cells, but not in primary keratinocytes. Finally, luteolin does not affect intracellular ATP production or viability. Appropriate formulations of luteolin and related flavones may be promising candidates to be developed into local and systemic treatments for Ps and other inflammatory skin diseases.

## Introduction

Keratinocytes represent the major cell type found in the epidermal layer of the skin. They are fully immunocompetent and can release inflammatory mediators such as interleukin (IL)-1, IL-6, IL-8, and vascular endothelial growth factor (VEGF), in response to immune and endocrine triggers [1]–[3]. Keratinocytes are involved in the pathogenesis of psoriasis (Ps), through their hyperproliferation and associated chronic inflammation [4]–[6]. With a prevalence of approximately 2–3% of the world’s population and typical lifetime duration of more than 30 years, the combined cost of Ps has a major impact on health care.

Tumor necrosis factor (TNF) plays a key role in the pathogenesis of Ps [7] and is produced by a number of cell types, including activated keratinocytes, T cells and mast cells [8]. TNF can trigger keratinocytes to produce IL-1 [9], which then stimulates both keratinocytes and mast cells to release IL-6 [10]. IL-6 then exerts autocrine actions and stimulates proliferation of cultured human keratinocytes [11]. TNF stimulates keratinocytes to release VEGF, which leads to dysregulated angiogenesis that contributes to the development of Ps-like dermatitis [2], [12]. In addition, TNF triggers activation of nuclear factor-kappa B (NF-κB), a transcription factor involved in inflammatory mediator production, which is upregulated in Ps lesional skin [13].

Since the role of TNF in Ps pathogenesis has become more recognized, anti-TNF biologic agents, such as etanercept, have been developed and shown to be more effective than traditional treatments for Ps, including methotrexate, psoralen plus ultraviolet A (PUVA), cyclosporine, and acitretin [8], [14]. Unfortunately, anti-TNF agents are also associated with increased risk of infections, and more recently blood malignancies [15], [16]. Other biologic agents have been introduced targeting IL-12, IL-17 and IL-23 [17], [18], but the long-term efficacy data remain unknown [19]. Therefore, the need for safe and effective long-term treatments for Ps is still of major importance.

Recently an increasing number of plant-derived molecules have shown positive effects in clinical trials in the treatment of inflammatory skin diseases [20], but the specific ingredients in the plant extracts are not well defined. Flavonoids, such as quercetin and luteolin, are natural compounds found in a number of plants with potent antioxidant and anti-inflammatory properties [21], [22]. Quercetin suppresses ultraviolet irradiation-induced expression of inflammatory cytokines IL-1β, IL-6, IL-8 and TNF in human keratinocytes [23]. Quercetin has also been shown to inhibit contact dermatitis and photosensitivity in humans [24]. On the other hand, the effects of luteolin on human keratinocytes have not been fully investigated, and its precise mechanism of action remains unknown.

In the present study, we examined effects of luteolin on activation of human cultured keratinocytes. We show that luteolin effectively inhibits TNF-induced production of inflammatory mediators IL-6, IL-8 and VEGF, as well as proliferation of human keratinocytes. Luteolin also decreases TNF-triggered activation of the transcription factor NF-κB at both the gene and protein levels. In addition, the mRNA expression of RELA (the gene encoding NF-κB p65 subunit) is elevated in human Ps skin. Our results suggest that luteolin is a promising candidate for development into effective treatment for inflammatory skin conditions, including Ps that is characterised by keratinocyte hyperproliferation and chronic inflammation.

## Materials and Methods

### Drugs and Reagents

Luteolin was purchased from Sigma-Aldrich (St. Louis, MO) and dissolved in DMSO. Recombinant human TNF was purchased from R&D Systems (Minneapolis, MN) and dissolved in double distilled water. The final concentration of DMSO was <0.1% and the pH was 7.4. Rabbit monoclonal antibodies against NF-κB p65 (D14E12) and β-actin (D6A8), and mouse monoclonal antibody against lamin A/C (4C11) were purchased from Cell Signaling (Danvers, MA).

### Human Keratinocytes

The immortalized human keratinocyte cell line HaCaT was used since it has been shown to be suitable for studies relavant to Ps [25], [26]. HaCaT keratinocytes were kindly provided by Dr. A. Slominski (University of Tennessee, Menphis, TN) and cultured in DMEM supplemented with 10% FBS and 1% penicillin/streptomycin (Sigma-Aldrich).

Adult normal human epidermal keratinocytes (NHEKs) purchased from Life Technologies (Carlsbad, CA) were cultured in EpiLife serum-free medium containing Human Keratinocyte Growth Supplement (Life Technologies).

### Cytokine Release by Enzyme-Linked Immunosorbent Assay (ELISA)

Keratinocytes (5×10^4^ cells per well) were seeded in 12-well plates (Becton Dickinson, Franklin Lakes, NJ) and allowed to grow overnight before stimulation with TNF (50 ng/mL, 24 h). In some experiments, cells were pretreated with luteolin; control cells were treated with 0.1% DMSO. IL-6, IL-8 and VEGF release were measured in supernatant fluids by ELISA using commercial kits from R&D Systems.

### Human Subjects

Punch skin biopsies (3 mm^3^) were collected from non-exposed lesional skin (back and gluteal) from Ps patients, who had not received any medication for 15 days prior to biopsy, and were free from any systemic allergic or inflammatory diseases, as well as healthy controls (n = 26). Biopsies were immediately placed in RNAlater solution (Ambion, Inc., Austin, Texas) and stored at −80°C. The Medical Ethics Committees of Attikon and A. Sygros Hospitals approved this protocol. The Declaration of Helsinki protocols were followed and patients provided their written informed consent. All human samples had no identifiers except for age and sex.

### RNA Isolation and Quantitative Real Time PCR (qRT-PCR)

Keratinocytes were pretreated with luteolin (10–100 µM, 30 min) before TNF stimulation (50 ng/mL, 6 h). Total RNA was extracted with an RNeasy Mini kit (Qiagen Inc., Valencia, CA). For human skin samples, total RNA was extracted using a Fibrous Tissue mini kit (Qiagen). An iScript cDNA synthesis kit (BioRad, Hercules, CA) was used for reverse-transcription of each sample. qRT-PCR was performed using Taqman gene expression assays (Applied Biosystems, Foster City, CA) for IL-6, IL-8, VEGF, NFKB1 and RELA. Samples were run using a 7300 Sequence Detector, according to TaqMan Gene Expression Assay instructions (Applied Biosystems). The mRNA expression was determined from standard curves run with each experiment. Relative mRNA levels were normalized to human GAPDH endogenous control (Applied Biosystems).

### NF-κB p65 Phosphorylation Assay

After luteolin pretreatment (10–100 µM, 6 h), HaCaT cells (2×10^6^ cells) were stimulated with TNF (50 ng/mL, 15 min). Phosphorylation of NF-κB p65 (serine 536) was detected by the PathScan Inflammation Sandwich ELISA kit (#7276, Cell Signaling) according to the instructions provided. Whole cell lysates were assayed at a protein concentration of 5 mg/mL. Absorbance was read at 450 nm using a LabSystems Multiskan RC microplate reader (Fisher Scientific, Cambridge, MA). Relative phospho-NF-κB p65 levels were normalized to control cells treated with 0.1% DMSO.

### Nuclear Translocation of NF-κB p65 by Western Blot Analysis

After luteolin pretreatment (100 µM, 6 h), HaCaT cells (2×10^6^ cells) were stimulated with TNF (50 ng/mL, 15 min). Cells were harvested and cytosolic and nuclear extracts were isolated using a NE-PER Nuclear and Cytoplasmic Extraction kit (Thermo Scientific, Rockford, IL). Antibody against lamin A/C was used to assess purity of cytosolic and nuclear extracts. Protein concentrations were determined using a BCA protein assay kit (Pierce Biotechnology, Rockford, IL). Aliquots of the protein extracts (each containing 20 µg of protein) were boiled for 5 min and electrophoresed on NuPAGE 4–12% Bis-Tris gel (Life Technologies). The resolved proteins were then transferred to PVDF membranes before antibody probing at room temperature using a SNAP i.d. system (Millipore, Billerica, MA). Antibody binding was detected using an Amersham ECL Prime detection kit (GE Healthcare, Buckinghamshire, UK). Densitometric analysis of Western blot bands was performed using Image J software downloaded from the NIH website (Bethesda, MD).

### NF-κB p65 DNA-binding Activity Assay

After luteolin pretreatment (10–100 µM, 6 h), HaCaT cells (10×10^6^ cells) were stimulated with TNF (50 ng/mL, 15 min). Cells were then harvested and cytosolic and nuclear extracts were isolated as described above. DNA-binding activity of NF-κB p65 was detected by the NF-κB (p65) Transcription Factor Assay Kit (#10007889, Cayman Chemical Co., Ann Arbor, MI). Cytosolic and nuclear extracts (each containing 10 µg of protein) were added to a 96-well plate coated with a specific double stranded DNA sequence containing the NF-κB response element. NF-κB was detected by addition of specific primary antibody directed against NF-κB followed by HRP-conjugated secondary antibody to provide a colorimetric readout at 450 nm. Relative NF-κB p65 DNA-binding activities in the cytosolic and nuclear extracts were normalized to control cells treated with 0.1% DMSO only.

### Cell Proliferation

HaCaT cells (2×10^5^ cells per well) were seeded in 6-well plates and treated with luteolin (10–100 µM) for up to 3 days. Cell proliferation was measured using the XTT (2, 3-Bis-(2-Methoxy-4-Nitro-5-Sulfophenyl)-2H-Tetrazolium-5-Carboxanilide)-based *in vitro* toxicology assay kit (Sigma-Aldrich). Absorbance at 450 nm is directly proportional to the number of live cells. Cell viability was determined by Trypan-blue (0.4%) exclusion.

### Intracellular ATP Measurement

Intracellular ATP content was measured to determine if luteolin has any effect on intracellular energy production. After luteolin incubation (10–100 µM) for 3 days, HaCaT cells (1×10^6^) were lysed and intracellular ATP content was determined using an ATP assay kit (Abcam, Cambridge, MA) according to instructions provided.

### Statistical Analysis

All experiments were performed in triplicates and were repeated at least three times (n = 3). Results are presented as mean ± SD. For *in vitro* experiments, data from stimulated and control samples were compared using the unpaired, two-tailed, Student’s *t*-test. For human skin gene expression of NFKB1 and RELA, results from Ps patients and controls were compared using the Mann-Whitney non parametric U-test. Statistical significance is denoted by * p<0.05, ** p<0.01, and *** p<0.001.

## Results

### Luteolin Inhibits IL-6, IL-8 and VEGF Production in HaCaT Keratinocytes

**Figure 1:**
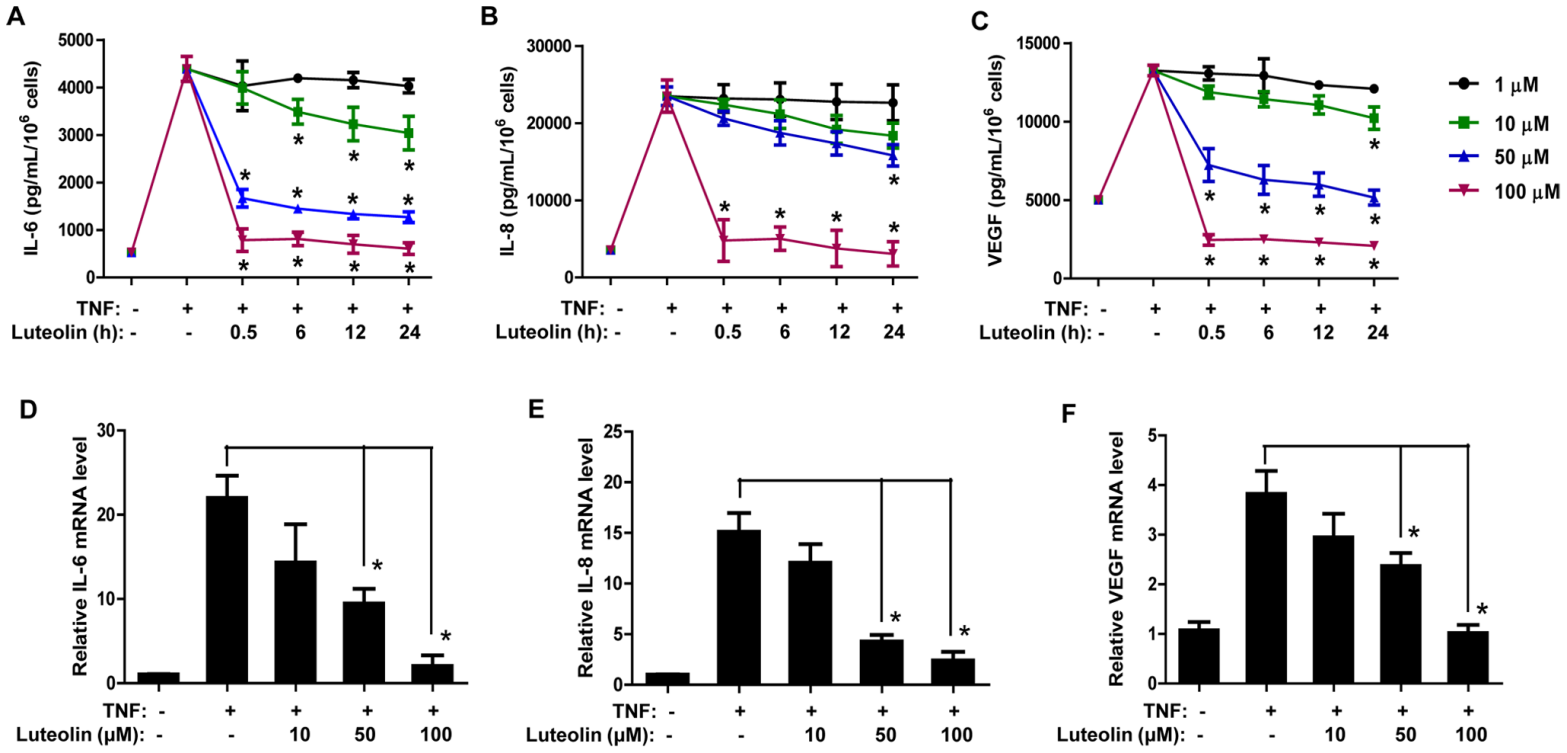
Luteolin inhibits IL-6, IL-8 and VEGF production from TNF-triggered HaCaT keratinocytes. HaCaT cells were pretreated with luteolin (1–100 µM) for various times as indicated and then stimulated with TNF (50 ng/mL, 24 h). Mediators in the supernatant fluids were measured by ELISA: (A) IL-6; (B) IL-8; (C) VEGF. The mRNA expression levels of (D) IL-6, (E) IL-8 and (F) VEGF were also determined after TNF stimulation (50 ng/mL, 6 h) by qRT-PCR (h = hours; n = 3, * p<0.05).

Stimulation of HaCaT cells with TNF (50 ng/mL, 24 h) significantly triggers release of IL-6, IL-8 and VEGF by 5-fold compared to control cells (Fig. 1A–C). Luteolin pretreatment (1–100 µM) inhibits mediator release in a concentration-dependent manner. Prolonged pretreatment does not increase the extent of inhibition, except for IL-6 release where inhibition by luteolin (10 µM) becomes more prominent with pretreatment time of 24 h as compared to 30 min (Fig. 1A). A higher luteolin concentration (100 µM) completely blocks IL-6, IL-8 and VEGF release.

Furthermore, the effect of luteolin on mRNA expression of IL-6, IL-8 and VEGF was examined. In HaCaT cells, TNF stimulation (50 ng/mL, 6 h) triggers a 20-fold increase in IL-6 mRNA level, a 15-fold increase in IL-8 mRNA level, and a 4-fold increase in VEGF mRNA level as compared to control cells (Fig. 1D–F). Pretreatment with luteolin (10–100 µM, 30 min) decreases mRNA expression of all three mediators, with complete inhibition achieved at 100 µM.

### Luteolin Decreases IL-6, IL-8 and VEGF Production in Normal Human Epidermal Keratinocytes (NHEKs)

**Figure 2:**
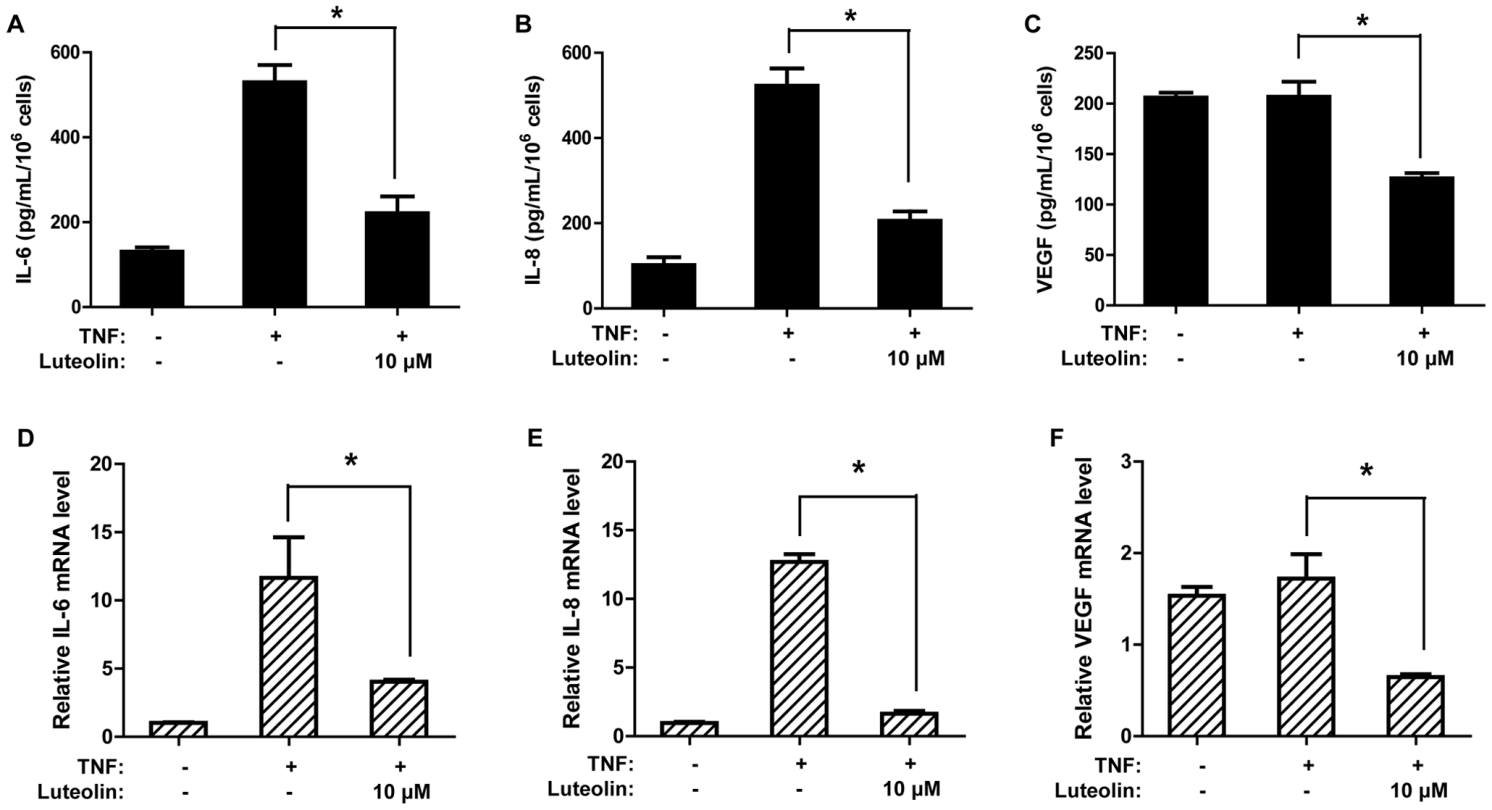
Luteolin decreases production of IL-6, IL-8 and VEGF in TNF-triggered primary NHEKs. NHEKs were pretreated with luteolin (10 µM, 30 min) before stimulation with TNF (50 ng/mL). Mediator release was measured at 24 h by ELISA: (A) IL-6; (B) IL-8; (C) VEGF. The mRNA expression levels were measured at 6 h by qRT-PCR: (D) IL-6; (E) IL-8; (F) VEGF (n = 3, * p<0.05).

Stimulation of NHEKs with TNF (50 ng/mL, 24 h) induces IL-6 and IL-8 release (Fig. 2A, B), but at a much lower level compared to HaCaT cells (Fig. 1A, B). However, TNF does not trigger VEGF release in NHEKs (Fig. 2C) as it does for HaCaT cells. Interestingly, NHEKs are more sensitive than HaCaT cells to the inhibitory effect of luteolin. Luteolin pretreatment (10 µM, 30 min) blocks TNF-triggered IL-6 and IL-8 release by more than 90%, and decreases basal VEGF production in NHEKs (Fig. 2C).

Similarly, TNF stimulation (50 ng/mL, 6 h) triggers about 12-fold increase in mRNA levels of IL-6 (Fig. 2A) and IL-8 (Fig. 2B) as compared to control cells. TNF does not stimulate VEGF mRNA expression in NHEKs (Fig. 2C). The mRNA expression of all three mediators is significantly decreased by pretreatment with a lower concentration of luteolin (10 µM, 30 min) (Fig. 2D–F) as compared to the concentration required (50 µM, 30 min) to achieve similar inhibition in HaCaT cells (Fig. 1D–F).

### Luteolin Decreases TNF-stimulated Activation of NF-κB

**Figure 3:**
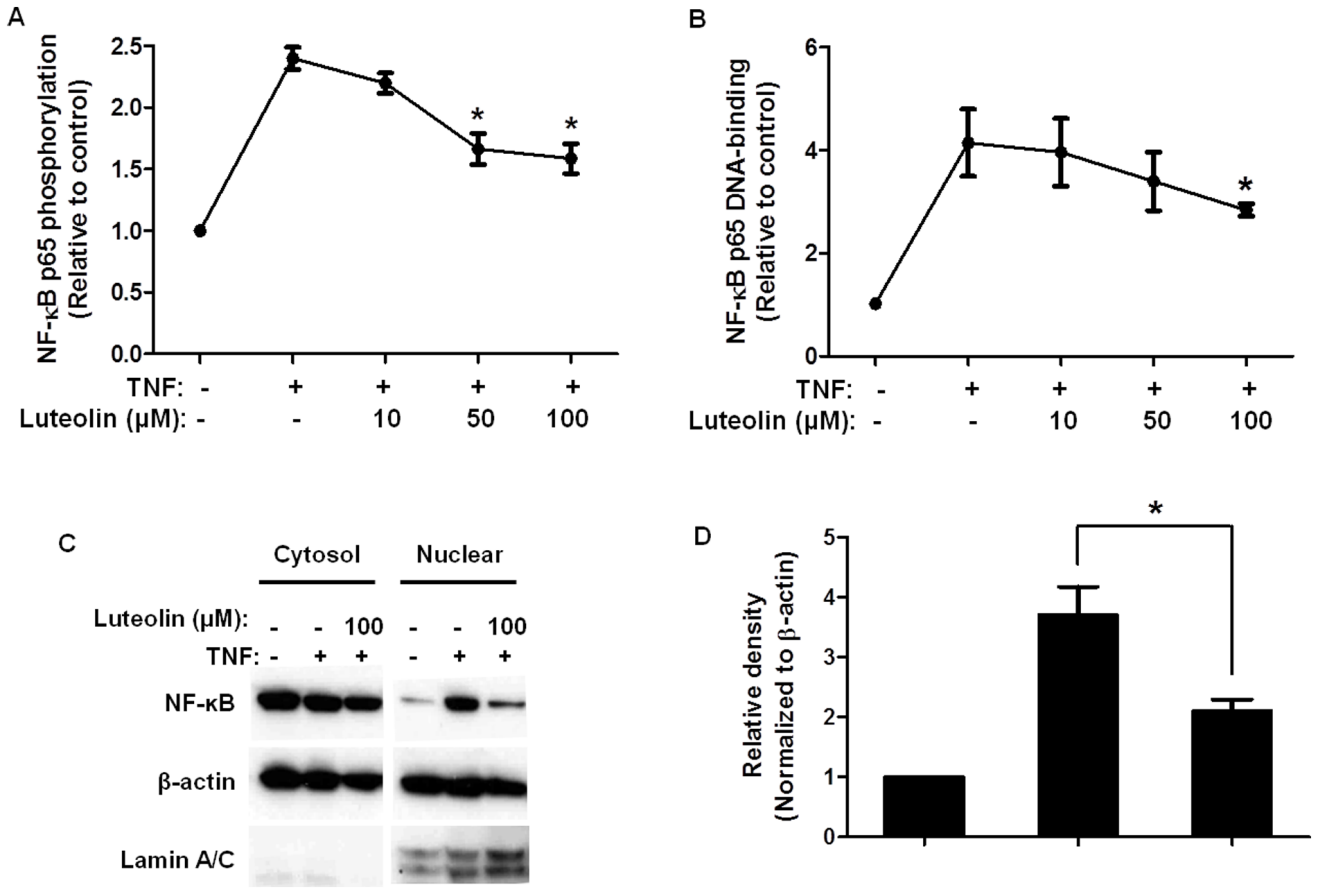
Luteolin reduces TNF-triggered NF-κB activation in HaCaT keratinocytes. HaCaT cells were incubated with luteolin (10–100 µM, 6 h) and then stimulated with TNF (50 ng/mL, 15 min). (A) TNF-triggered phosphorylation of NF-κB p65 was detected using a Multi-Target Sandwich ELISA kit and is presented as fold changes relative to control. (B) NF-κB p65 DNA-binding activity in the nuclear extract was examined and expressed as fold changes relative to control. (C) Nuclear translocation of NF-κB p65 was determined using Western blot and quantified by densitometry (D). The Western blot shown in (C) was representative of 3 independent experiments. (n = 3, * p<0.05).

Stimulation of HaCaT cells with TNF (50 ng/mL, 15 min) rapidly causes NF-κB p65 phosphorylation, which is significantly reduced by pretreatment with luteolin (50 and 100 µM, 6 h) by 50% compared to control cells (Fig. 3A). TNF stimulation (50 ng/mL, 15 min) also increases NF-κB p65 DNA-binding activity in the nucleus, which is significantly decreased by 22% and 51% with luteolin pretreatment (50 and 100 µM, respectively, 6 h) (Fig. 3B). This is further supported by Western blot analysis showing significant reduction of NF-κB p65 nuclear translocation in luteolin (100 µM)-pretreated cells (Fig. 3C, D). To verify that there was no cross-contamination of nuclear and cytosolic extracts, a nuclear protein lamin A/C, was probed and shown to be present in the nuclear extract only, and absent in the cytosolic extract (Fig. 3C).

### Luteolin Reduces TNF-triggered mRNA Expression of NFKB1 and RELA

**Figure 4:**
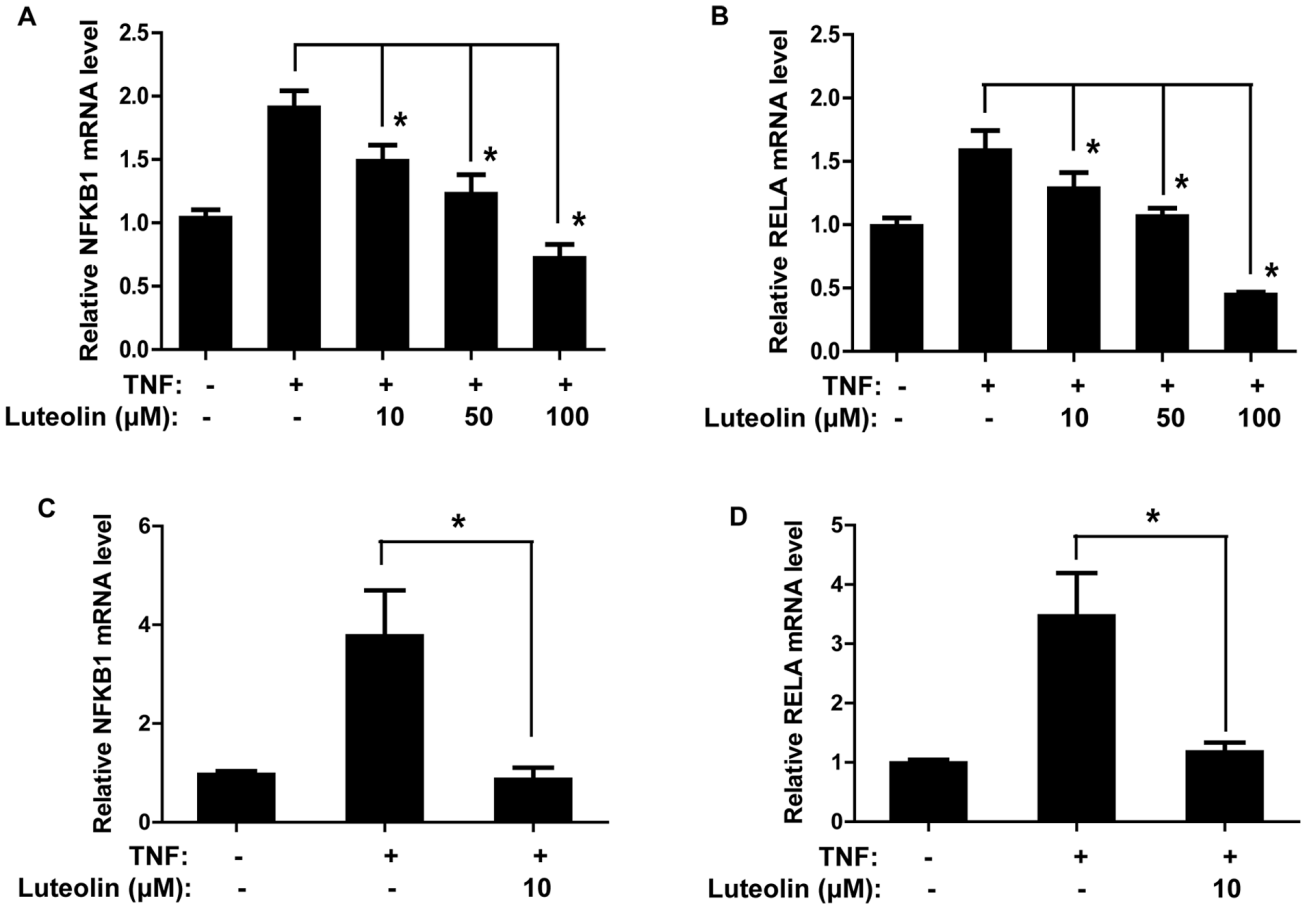
Luteolin reduces TNF-stimulated mRNA expression of NFKB1 (NF-κB p50) and RELA (NF-κB p65) in keratinocytes. The effect of luteolin on mRNA expression levels of two genes encoding NF-κB subunits were investigated using qRT-PCR. HaCaT cells were pretreated with luteolin (10–100 µM, 30 min) before stimulation with TNF (50 ng/mL, 6 h): (A) NFKB1, (B) RELA. NHEKs were pretreated with luteolin (10 µM, 30 min) before stimulation with TNF (50 ng/mL, 6 h): (C) NFKB1, (D) RELA. (n = 3, * p<0.05).

We also studied for the first time, to our knowledge, the effect of luteolin on mRNA expression of two genes encoding two different NF-κB subunits, NFKB1 (encoding NF-κB p50 subunit) and RELA (encoding v-rel reticuloendotheliosis viral oncogene homolog A or NF-κB p65 subunit). TNF-triggers slight increases of NFKB1 and RELA mRNA levels in HaCaT cells, which are significantly reduced by pretreatment with luteolin (10–100 µM, 30 min) in a concentration-dependent manner (Fig. 4A, B).

In NHEKs, TNF triggers more than 3-fold increase of NFKB1 and RELA mRNA levels compared to control cells (Fig. 4D, E), which is a more prominent increase than that observed in HaCaT cells. Luteolin pretreatment at a low concentration (10 µM, 30 min) effectively decreased mRNA expression of both NFKB1 and RELA to the basal level (Fig. 4D, E).

### Gene Expression of RELA is Increased in Ps Skin

**Figure 5:**
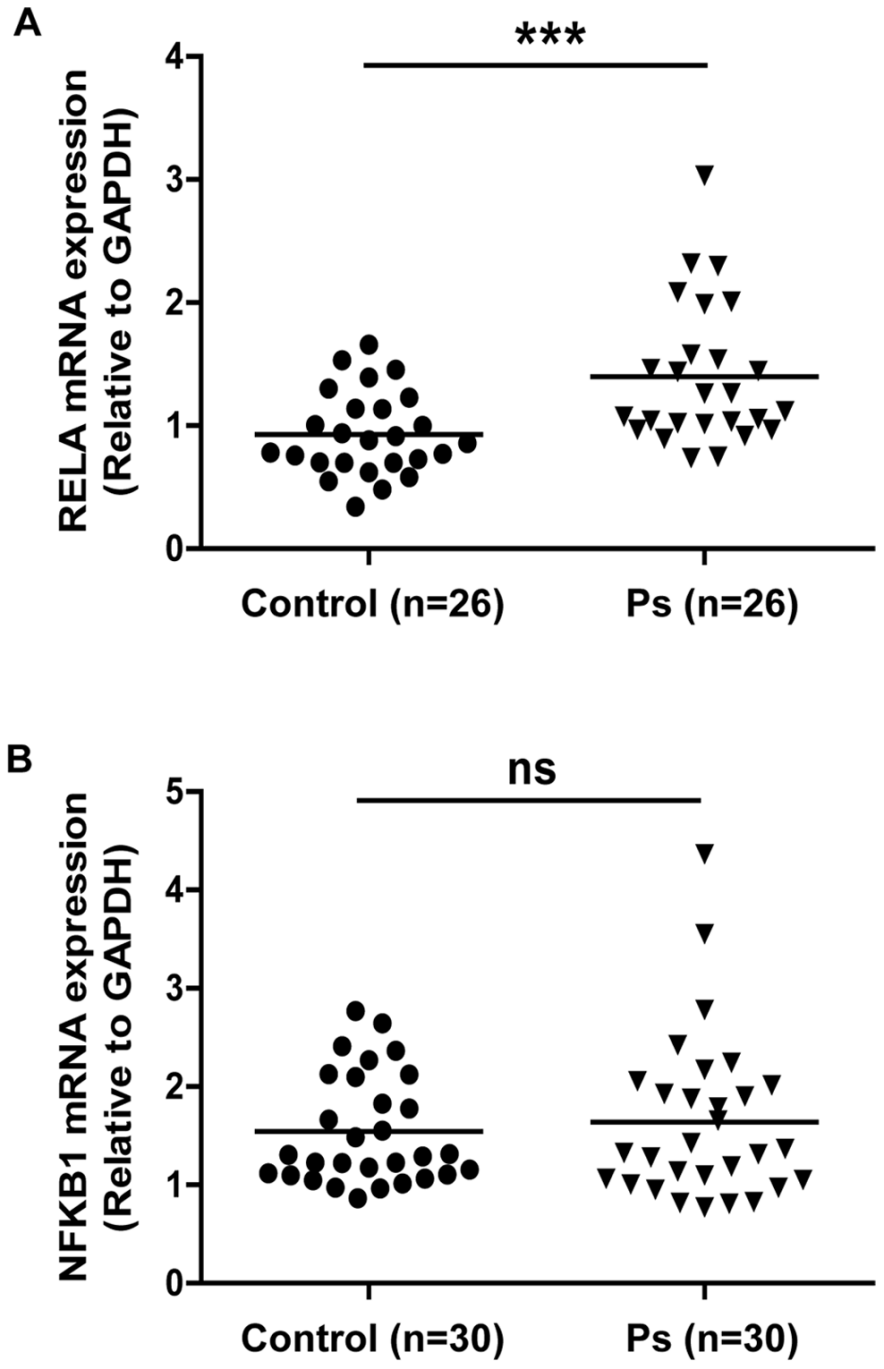
Gene expression of RELA is increased in Ps lesional skin. Total RNA was extracted from Ps lesional skin biopsies and qRT-PCR was performed for mRNA expression levels of (A) RELA and (B) NFKB1, which were normalized to human GAPDH as an endogenous control (ns = not significant, *** p<0.001).

We further investigated the mRNA expression of NFKB1 and RELA in skin biopsies obtained from Ps patients (n = 26–30). There was no statistical difference in the mean age between Ps patients and controls. In Ps lesional skin, RELA (p = 0.0007, Fig. 5A) mRNA expression increases by 1.5-fold as compared to healthy controls. In contrast, there is no statistically significant difference in NFKB1 mRNA expression between Ps patients and controls (Fig. 5B).

### Luteolin Reduces Keratinocyte Proliferation without Affecting Intracellular ATP Content

**Figure 6:**
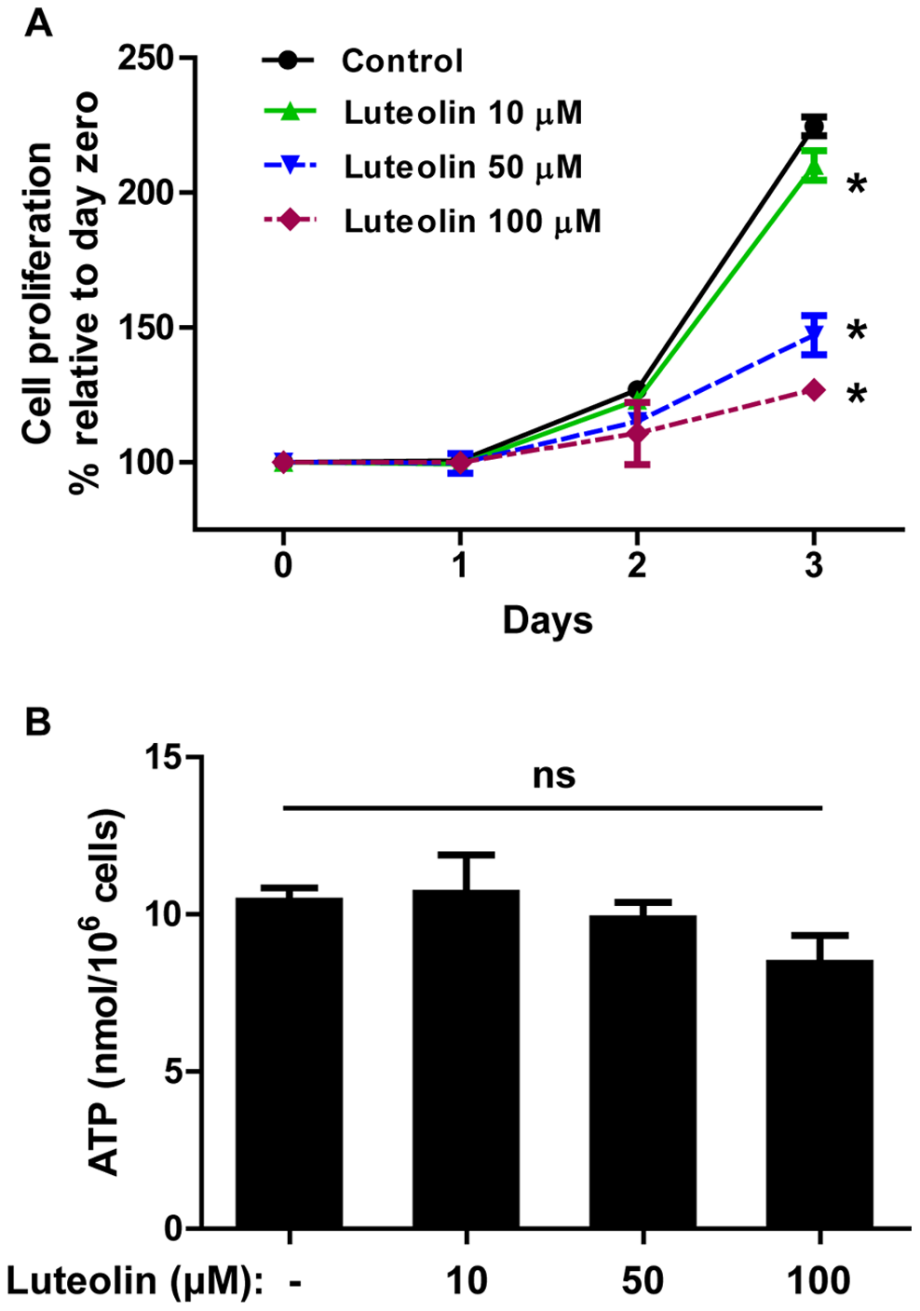
Luteolin decreases HaCaT keratinocyte proliferation without affecting ATP production. HaCaT cells were incubated with luteolin (10–100 µM) for up to 3 days. (A) Cell proliferation was examined using a XTT-based assay. (B) Intracellular ATP content was determined using an ATP assay kit (n = 3, ns = not significant, * p<0.05).

We examined the effect of luteolin on keratinocyte proliferation using the XTT-based assay. The proportion of XTT reduction is directly proportional to the number of viable cells in culture. Luteolin (10 µM) significantly reduces keratinocyte proliferation compared to control cells after 3-day incubation (Fig. 6A). The inhibition is more dramatic with luteolin at 50 µM and 100 µM. It is important to note that cell viability remains above 90%. Interestingly, luteolin has no effect on proliferation of primary NHEKs (data not shown). Noteworthy, cytokine gene and protein expression are measured at 6 and 24 h, respectively, when there is no effect on cell proliferation by luteolin.

In order to investigate if luteolin has any effect on cellular metabolic activity, we measured intracellular ATP content. In HaCaT cells, although luteolin incubation (100 µM, 3 days) slightly reduced intracellular ATP content (13%, Fig. 6B) compared to control cells, this was not statistically significant.

## Discussion

Ps is a debilitating disease involving increased inflammatory cytokine production and keratinocyte hyperproliferation [8], [27]. Despite success in Ps treatment with anti-proliferative and anti-TNF therapies, many patients experience serious side effects and drug resistance [8], [28]. New biological therapies have revolutionized the treatment for Ps, but none of these treatments address release of cytokines or keratinocyte proliferation. There is also a need for less invasive approaches, especially for patients with mild Ps symptoms. Here we show that the natural flavonoid luteolin significantly inhibits TNF-triggered production of inflammatory mediators (IL-6, IL-8 and VEGF) from human keratinocytes, and also decreases activation of the transcription factor NF-κB. Moreover, we report for the first time, to the best of our knowledge, luteolin reduces mRNA expression of two genes (NFKB1 and RELA) encoding two subunits (p50 and p65, respectively) in the NF-κB protein complex, that is important for NF-κB activation.

The mediators TNF, IL-17, IL-6, IL-8, CCL2 and VEGF play a pivotal role in the initiation and progression of chronic inflammation [8]. Previously, it has been reported that the flavonol quercetin has the ability to actively accumulate in the nucleus and modify the activity of numerous transcription factors involved in the production of a number of inflammatory mediators [29]. In our studies, luteolin readily enters the cells (unpublished data). As a structurally-related flavonoid to quercetin, we suggest that luteolin might have similar actions and regulate gene transcription inside the nucleus.

We show that luteolin decreases TNF-triggered phosphorylation, nuclear translocation and DNA-binding activity of the transcription factor NF-κB, which is an inducible transcription factor constitutively expressed in HaCaT cells [30]. TNF induces NF-κB activation, which sets up an autocrine signaling loop that leads to further TNF secretion and sustained NF-κB activation [31]. Previous studies also suggest that the NF-κB pathway plays an essential role in Ps progression [32], [33], where there is marked elevation of active phosphorylated NF-κB p65 in Ps lesional skin [13]. Luteolin could be inhibiting inflammatory mediator production via blockade of NF-κB activation. However, it is also possible that luteolin acts on signaling molecules upstream of NF-κB activation, such as the mammalian target of rapamycin (mTOR) that governs cell size, growth, and metabolism [34]. Recent papers provided early evidence that the mTOR pathway may be involved in activation and proliferation of keratinocytes [35], [36]. Experiments are currently underway in our lab to determine potential effects of luteolin on the mTOR pathway (unpublished data).

Apart from inhibiting NF-κB protein activation, we also report for the first time that luteolin decreases TNF-induced mRNA expression of two genes (NFKB1 and RELA) encoding subunits (p50 and p65, respectively) in the NF-κB protein complex. Our results further show that mRNA expression of RELA is significantly increased in Ps lesional skin. Being able to inhibit NF-κB activation at both the mRNA and protein levels in keratinocytes, luteolin is likely to offer benefits in treating Ps, where there is sustained NF-κB activation. Previously, it has been shown that skin biopsies from Ps patients treated with the anti-TNF therapy etanercept for 1, 3 and 6 months showed a significant reduction of NF-κB [13] supporting that blockade of TNF activity results in NF-κB downregulation. In our results, although the differences of RELA skin mRNA expression level between Ps patients and controls are modest, chronic Ps could downregulate these NF-κB-related genes. In addition, the 15-day wash-out period before taking biopsies from Ps patients might not be long enough for full recovery of NF-κB gene expression.

Ps is also characterized by increased number of lesional skin mast cells [37], the only known cell type that rapidly secretes preformed TNF [38], [39], which could stimulate keratinocytes. Luteolin has been shown to inhibit mast cell degranulation [40] and cytokine release [41]. Luteolin also inhibits mast cell-dependent stimulation of activated T cells [42], [43], a key feature in Ps pathogenesis [8]. Moreover, the marked inhibition of IL-6 gene expression and secretion by luteolin is particularly important since IL-6 is required to drive maturation of Th17 cells [44], which are also involved in Ps pathogenesis [27]. Thus, luteolin could antagonize the activation of and interactions between multiple cell types involved in Ps, including keratinocytes, mast cells and T cells. In addition, increased keratinocyte proliferation is a characteristic of Ps dermis. Here we demonstrate that luteolin significantly decreases HaCaT, but not normal keratinocyte proliferation without causing apparent cell death.

The flavonoid luteolin is lipid-soluble and could be developed into topical formulations that easily penetrate the skin. Luteolin is generally safe [45]–[47], and can even protect against chemically-induced hepatotoxicity [48] and nephrotoxicity [49]. Given that flavonoids also have anti-cancer properties [50], [51], luteolin could be used together with anti-TNF agents both for potential synergism and for protection against the cancer-inducing side effects of such agents.

In conclusion, our findings show that the natural flavonoid luteolin inhibits the production of inflammatory mediators IL-6, IL-8 and VEGF from TNF-triggered human keratinocytes. We also suggest for the first time, to the best of our knowledge, luteolin decreases NF-κB activation at both the transcriptional and translational levels. In addition, luteolin decreases keratinocyte proliferation without affecting viability and intracellular ATP production. Hence, luteolin has the potential to be developed into safer and more effective alternative therapies for the treatment of inflammatory skin diseases, including Ps.
